# Disorders of serum omega-3 fatty acid composition in dialyzed patients, and their associations with fat mass

**DOI:** 10.1080/0886022X.2017.1295870

**Published:** 2017-03-06

**Authors:** Małgorzata Sikorska-Wiśniewska, Adriana Mika, Tomasz Śledziński, Sylwia Małgorzewicz, Piotr Stepnowski, Bolesław Rutkowski, Michał Chmielewski

**Affiliations:** a Department of Nephrology, Transplantology and Internal Medicine, Medical University of Gdańsk, Gdańsk, Poland;; b Department of Environmental Analysis, Faculty of Chemistry, University of Gdańsk, Gdańsk, Poland;; c Department of Pharmaceutical Biochemistry, Medical University of Gdańsk, Gdańsk, Poland;; d Department of Clinical Nutrition, Medical University of Gdańsk, Gdańsk, Poland

**Keywords:** Omega-3 long-chain polyunsaturated fatty acids, hemodialysis, peritoneal dialysis, chronic kidney disease, lipid disorders

## Abstract

Patients with chronic kidney disease (CKD) are at increased risk of cardiovascular mortality. Lipid disorders, a constant feature of CKD, might contribute to this state. The aim of this study was to evaluate n-3 polyunsaturated fatty acids (PUFA) composition in CKD patients treated with dialysis, in comparison to the general population and to assess possible associations between the n-3 PUFA profile and anthropometric variables. Thirty-three prevalent dialysis patients were studied and compared with an age- and sex-adjusted control group of 22 patients. Fatty acid composition in serum was analyzed by gas chromatography with a mass spectrometer detector (GC-MS) and anthropometric measures were assessed by bioimpedance spectroscopy. The fatty acid profile of dialyzed patients was characterized by a significantly lower percentage content of n-3 PUFA. For α-linolenic acid (ALA), it was 0.21 ± 0.09% in dialysis patients versus 0.33 ± 0.11% in the control group (*p* < .001). For eicosapentanoic acid (EPA), 0.59 ± 0.23% versus 1.15 ± 0.87% (*p* < .001), and for docosahexaenoic acid (DHA) 1.11 ± 0.50% versus 1.75 ± 0.87% (*p* < .001), respectively. The amount of n-3 PUFA decreased with time on dialysis and it correlated positively with body fat mass. For DHA, this correlation was *r* = .48 (*p* < .01) and for EPA *r* = .40 (*p* < .05). Patients with CKD have a relatively low content of n-3 PUFA which may contribute to their high cardiovascular risk. Patients with a higher content of body fat are characterized by a favorable fatty acid composition.

## Introduction

Cardiovascular disease (CVD) is the leading cause of death in patients with chronic kidney disease (CKD), including among the dialysis population^1^. Despite of the progress in the management of renal replacement therapy, mortality remains very high and survival has not improved in last years. Cardiovascular mortality is several times higher, as compared to the general population. This high cardiovascular burden results from many risk factors.

Dyslipidemia is one of the major ones in the general population. Patients on hemodialysis (HD) and peritoneal dialysis (PD) maintenance exhibit many disturbances of serum lipid profile^2^. The most profound ones, low concentration of high-density lipoprotein (HDL) cholesterol and hypertriglyceridemia, are acknowledged CVD risk factors in the general population^3^^,^^4^. Similarly, they might promote the process of atherosclerosis and contribute to high cardiovascular mortality rate in dialysis patients. However, the exact contribution of dyslipidemia to atherosclerosis in dialysis patients remains unclear. Little is known about disorders of other lipid fractions such as fatty acids in this patient population. The n-3 polyunsaturated fatty acids (PUFA) compose a group of fatty acids characterized by a double bond at the third carbon atom from the end of the carbon chain. The major representatives of this group of PUFA are: α-linolenic acid (ALA 18:3n-3) derived mainly from plant oils, as well as eicosapentaenoic acid (EPA 20:5n-3) and docosahexaenoic acid (DHA 22:6n-3) that are found in marine oils. Numerous clinical and epidemiological studies have shown that these PUFA provide cardiovascular protection in the general population, although studies demonstrating lack of association between n-3 PUFA and CVD also exist^5^^,^^6^. N-3 PUFA decrease oxidative stress and act as anti-inflammatory agents by decreasing the production of pro-inflammatory cytokines, eicosanoids and the expression of adhesion molecules^7^. They are also thought to have anti-aggregation and anti-atherosclerotic effects. The issue of n-3 PUFA content and metabolism in CKD has been sparsely studied^8^. Therefore, the primary objective of this study was to evaluate the n-3 PUFA composition in a dialysis population in comparison to control subjects with no kidney insufficiency. Secondary purpose was to seek for potential associations between the n-3 PUFA profile and the clinical and anthropometric features of the studied patients.

## Material and methods

The study included 33 dialysis patients (23 treated with PD, 10 with HD), as well as an age- and sex-adjusted control group of 22 subjects without CKD. Plasma samples were collected after an overnight fast, and stored at −80 °C until analyzed.

Circulating levels of high-sensitivity C-reactive protein (CRP), albumin, total and HDL cholesterol as well as triglycerides were analyzed using certified methods at the Central Clinical Laboratory of the University Clinical Centre in Gdansk. Body fat and lean body mass was evaluated with the use of multifrequency bioimpedance analysis (BIA; Fresenius, Bad Homburg, Germany).

Protocol of the study received approval from the Local Bioethics Committee at the Medical University of Gdansk.

Total lipids were extracted from whole serum samples in a chloroform–methanol mixture (2:1, v/v) following the method by Folch et al.^9^, as described previously^10^. In brief, lipid extracts were dried by evaporation under a stream of nitrogen. Each sample was hydrolyzed with 1 mL of 0.5 M KOH in methanol at 90 °C for 3 h. The mixture was acidified with 0.2 mL of 6 M HCl and then 1 mL of water was added. Non-esterified fatty acids (FA) were extracted three times with 1 mL of *n*-hexane, and evaporated to dryness in a stream of nitrogen. FA methyl esters (FAME) were prepared using 1 mL of 10% boron trifluoride-methanol solution at 55 °C for 90 min. One milliliter of water was added to the reaction mixture and FAME were extracted three times with 1 mL of *n*-hexane and the solvent was evaporated. FAME were analyzed with GC-EI-MS QP-2010 SE (Shimadzu Corporation, Kyoto, Japan). FAME were separated on a 30 m; 0.25 mm i.d., Rtx-5MS capillary column (film thickness 0.25 μm). The column temperature was programed from 60 to 300 °C at a rate of 4 °C/min with helium as the carrier gas at a column head pressure of 60 kPa. For ionization of FAME, the electron energy was 70 eV. The internal standard was 19-methylarachidic acid. The n-3 PUFA amounts were expressed as percentages of total fatty acids by weight.

Results are expressed as mean and standard deviation or median and interquartile range, as appropriate. The assumption of normality was verified with the Kolmogorov–Smirnov test. A *p* values <.05 was considered to be statistically significant. Comparisons between two groups were assessed with a Student’s *t*-test, or a Mann–Whitney test, as appropriate. To assess correlations among the evaluated variables, Pearson’s correlation coefficient (*r*) was used. Statistical processing of the results was performed with the use of the statistical software Statistica PL version 10.0 (StatSoft, Kraków, Poland).

## Results

**Figure 1.**
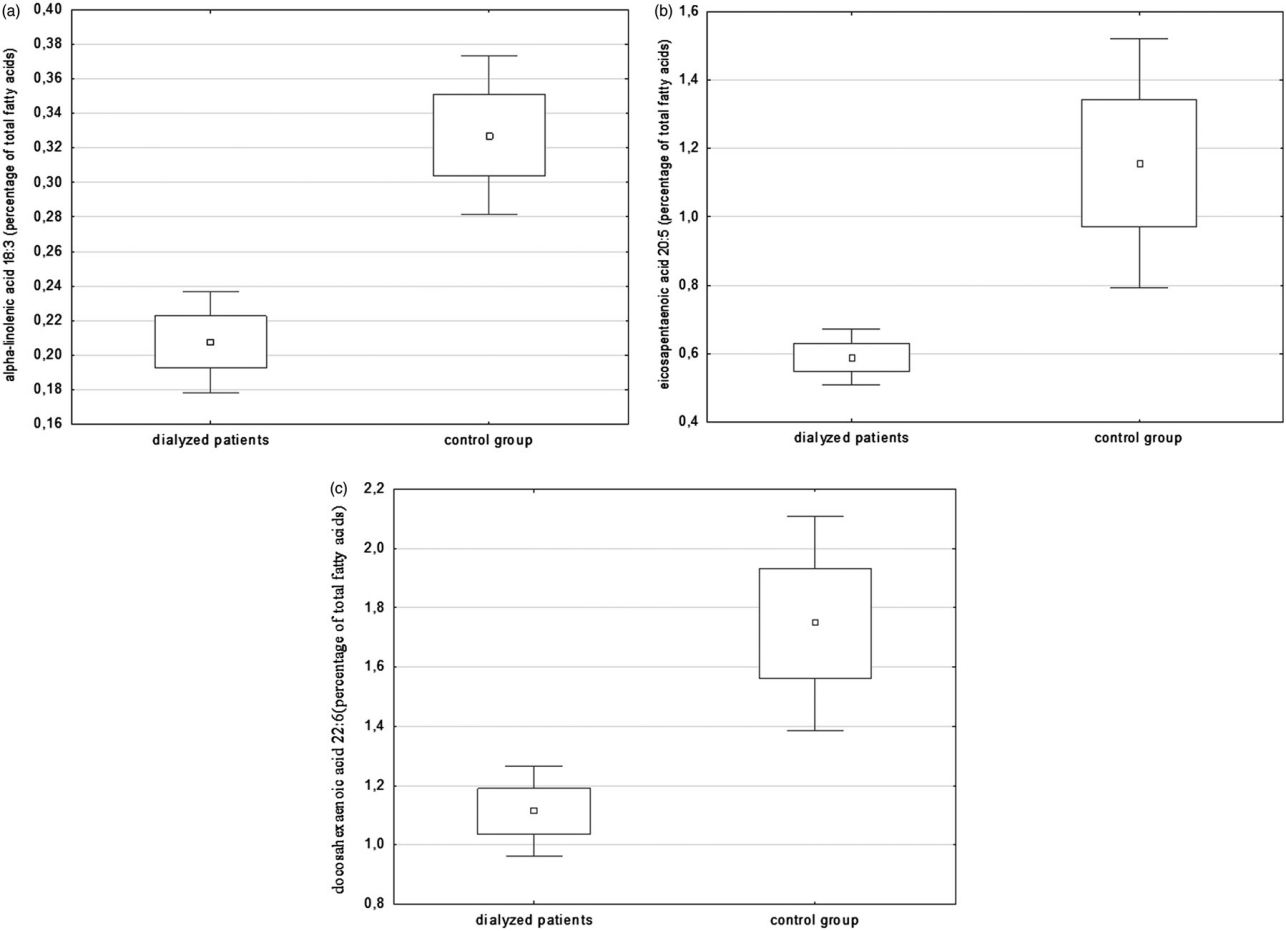
The percentage content of (a) ALA, (b) EPA and (c) DHA of dialysis patients and controls.

The dialysis group of 33 patients consisted of 23 treated with PD and 10 with HD (mean age 55.8 years, 18 men). The dialysis vintage was 6 (6–14) months in PD patients and 26.5 (6–60) months in HD subjects. The dialyzed group was evaluated in comparison to 22 controls without kidney insufficiency (mean age 53.5 years, 12 men). The analysis of fatty acid profile in dialyzed patients indicated a significantly lower content of n-3 PUFA in serum, in comparison to controls with no kidney disease. For ALA, it was 0.21 ± 0.09% in dialysis patients versus 0.33 ± 0.11% in controls (*p* < .001) (Figure 1(a)). For EPA, 0.59 ± 0.23 versus 1.15 ± 0.87 (*p* < .001), respectively (Figure 1(b)) and for DHA 1.11 ± 0.50 versus 1.75 ± 0.87 (*p* < .001), respectively (Figure 1(c)). Concentrations of plasma DHA and EPA were strongly correlated. This relationship was much weaker in the dialysis population than in the control group (*r* = .39, *p* < .05 versus *r* = .72, *p* < .001). Additionally the content of n-6 PUFA was examined (Table 1). The dialyzed group had a lower content of total plasma n-6 PUFA, including the content of arachidonic acid. We observed higher concentration of oleic acid (monounsaturated fatty acid) in dialysis population when compared to control group. There were no differences regarding n-3 PUFA or n-6 PUFA between the two dialysis modalities.

**Table 1.** Fatty acids amounts in patients treated with peritoneal dialysis, hemodialysis and controls.

Fatty acids amount	PD patients(*n* = 23)	HD patients(*n* = 10)	Control group(*n* = 22)
n-3 PUFA, %	3.56 ± 0.54	3.41 ± 0.63	5.08 ± 1.72
ALA, %	0.22 ± 0.09	0.19 ± 0.08	0.33 ± 0.11
EPA, %	0.61 ± 0.24	0.54 ± 0.26	1.16 ± 0.87
DHA, %	1.1 ± 0.4	1.2 ± 0.5	1.75 ± 0.87
n-6 PUFA, %	3.89 ± 1.19	4.03 ± 1.04	6.25 ± 1.64
Arachidonic acid, %	3.71 ± 1.17	3.84 ± 1.04	6.01 ± 1.61
Oleic acid, %	28.44 ± 3.28	26.34 ± 3.52	23.70 ± 2.96

Fatty acids amounts were expressed as percentages of total fatty acids by weight; *p* < .05 for all the variables for differences between dialysis patients and controls.

**Figure 2.**
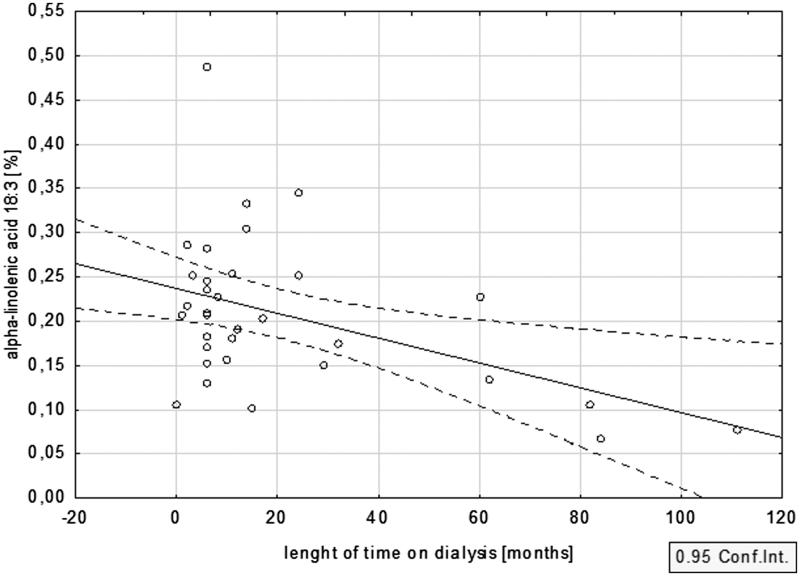
Relationship between dialysis vintage and the percentage content of ALA.

The percentage amount of n-3 PUFA decreased with time on dialysis. This inverse relationship with dialysis vintage was especially evident for ALA (*r*= −0.45; *p* < .05) (Figure 2), and somewhat weaker for EPA and DHA (*r*= −.17, *p* = .36 and *r*= −.21, *p* = .25, respectively).

The n-3 PUFA content did not show any correlation with the other parameters of lipid profile, i.e. with the total, LDL or HDL cholesterol, as well as with triglyceride concentration. Similarly, no relationship was found to parameters of inflammation and nutrition, i.e. CRP and albumin.

**Figure 3.**
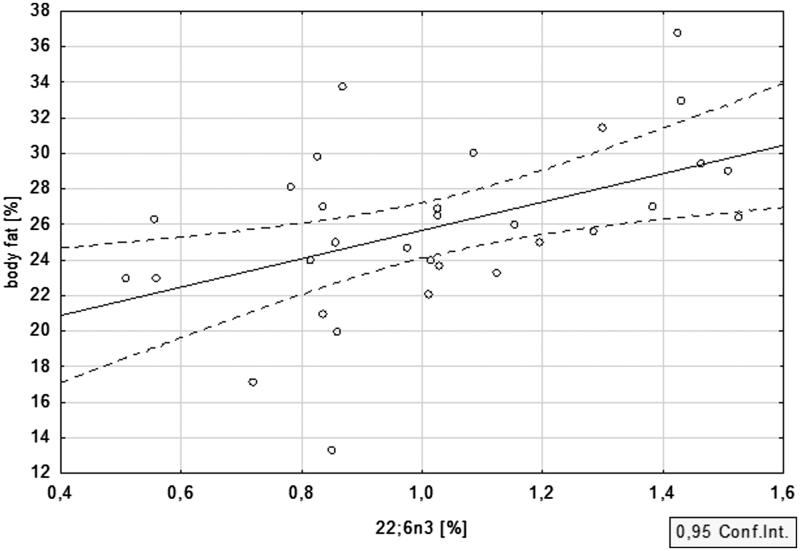
Relationship between body fat and DHA in dialysis patients.

**Figure 4.**
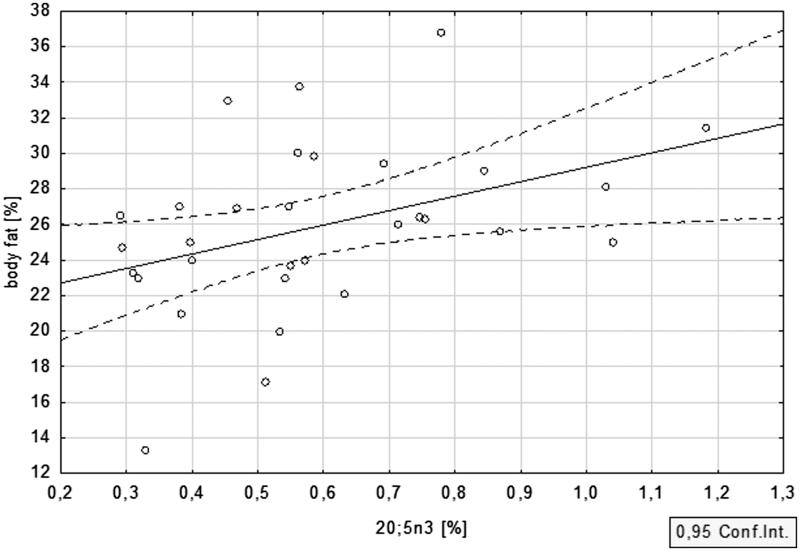
Relationship between body fat and EPA in dialysis patients.

Anthropometric features of the studied population were evaluated with the use of multifrequency bioimpedance. The percent of body fat equalled 25.5 ± 5.5 in PD patients, and 24.9 ± 3.2 in HD subjects, while the mean lean body mass was 55.7 ± 10.2 and 58.7 ± 7.9 kg, respectively. Strong correlation between n-3 PUFA and body fat in the CKD cohort was revealed. For DHA, this correlation was equal to *r* = .48; *p* = .006 (Figure 3) and for EPA *r* = .40; *p* = .03 (Figure 4). ALA displayed a similar association, although it did not reach statistical significance (*r* = .19, *p* = .30). The associations between n-3 PUFA and lean body mass were not statistically significant.

## Discussion

In the present study, in dialysis patients, we found a significantly lower content of all three major n-3 PUFA, as compared to controls without kidney insufficiency. The n-3 PUFA content declined with time on dialysis, being lowest in patients with the longest dialysis vintage. Moreover, a strong positive association between marine n-3 PUFA and fat mass was demonstrated.

Patients treated with dialysis are burdened with an exceptionally high risk of cardiovascular complications. The major factors responsible are typically divided into traditional and non-traditional. Dyslipidemia is an example of the first group, while persistent inflammation and wasting are important representatives of the latter. The n-3 PUFA are believed to impact both groups. Studies in the general population reveal that n-3 PUFA have the potential to lower TG concentration and to increase HDL cholesterol^11^. This improvement in the lipid profile might be especially important in CKD patients, in whom hypertriglyceridemia and low HDL cholesterol are common^2^. Persistent low-grade inflammation is another typical feature of CKD^12^. It is regarded as a significant contributor to increased cardiovascular and all-cause mortality of dialysis patients. Multiple experimental studies have shown that addition of n-3 PUFA reduces the inflammatory responses effectively by down-regulating or inhibiting expression of pro-inflammatory cascade compounds^13^. Persistent inflammation is an important contributor to wasting and sarcopenia, common and deleterious complications of CKD. Sarcopenia constitutes a significant and independent risk factor for mortality in both incident and prevalent dialysis patients^14^. Since n-3 PUFA incorporate into the membranes of skeletal muscles, exerting anti-inflammatory and anabolic effects, they are believed to protect from sarcopenia.

There is quite a number of studies demonstrating benefits from n-3 PUFA supplementation in dialysis patients. Kutner et al.^15^ showed that patients who reported higher fish consumption were ∼50% less likely to die during the 3-year observation period. In the work by Hamazaki et al.^16^, high DHA was independently associated with decreased mortality risk in HD subjects. In a large and representative cohort of incident HD patients from USA, there was a significant and independent inverse relationship between long-chain n-3 PUFA and the risk of sudden cardiac death^17^. Finally, in a study by Svensson et al.^18^, n-3 PUFA supplementation reduced the number of myocardial infarctions in HD subjects.

In the present evaluation, the n-3 PUFA content turned out to be significantly lower in dialysis patients, as compared to controls. This is in accordance with previous studies^19–21^. Furthermore, the n-3 PUFA tended to decrease with time on dialysis. Several factors might contribute to this altered FA composition: loss of appetite and/or dietary restrictions, presence of co-morbidities, loss of n-3 PUFA into the dialysate, etc. Friedman et al.^22^ showed that dietary fish consumption in dialysis patients is much lower than recommendations of the American Heart Association. There are few studies that estimate the influence of dialysis procedure on fatty acid composition. Dolegowska et al.^23^ demonstrated the effect of HD on the composition of fatty acid fractions of phospholipids in erythrocyte membranes. Friedman et al.^24^ measured the effect of a single-standard HD treatment on plasma and erythrocyte n-3 PUFA levels in chronic dialysis patients. The results of this study showed that the HD procedure induced acute increases in long-chain n-3 PUFA content in the blood that according to the authors, might explain why malignant cardiac arrhythmias occur relatively seldom during HD.

Although a decreased content of n-3 PUFA in dialysis patients in comparison to controls is a rather constant finding, the exact levels differ among various parts of the world. The percentage content of n-3 PUFA observed in this study is among the lowest recorded, much lower than reported in Japan^16^, although similar to the one observed among the US HD patients^25^. The most probable explanation for this diversity is the difference in dietary habits, including sea-food consumption, among countries and regions.

Apart from the n-3 PUFA from the marine sources, we studied the blood content of ALA, a n-3 PUFA derived from plant sources, as: green leafy vegetables, walnuts, soybean and canola oils. Mounting evidence demonstrates that ALA content and consumption relates to beneficial cardiovascular outcomes in a similar way as that of marine n-3 PUFA^26^. There are few studies in which ALA content has been evaluated in dialysis population. Dessi et al.^21^ measured ALA content, among other fatty acids, in plasma and erythrocyte phospholipids. ALA plasma levels were similar in patients on HD and in the control group, but they were reduced in erythrocyte membranes of HD patients. According to the authors, the reduction might be due to conversion of ALA to EPA and DHA or to dietary restrictions, as vegetables and nuts, the primary source of ALA, are also rich in potassium. Yerlikaya et al.^20^ measured fatty acid composition in plasma of patients on continuous ambulatory PD, and found ALA as well as other n-3 PUFA levels to be significantly lower, compared to the healthy control subjects. The present findings in our study may suggest that ALA follows a similar pattern to marine n-3 PUFA, with significantly lower levels in comparison to controls, and with a further decline during dialysis treatment. The clinical significance of these results require further studies.

The present study demonstrated possible association between the n-3 PUFA and fat mass. This is a novel finding, not evaluated in previous studies. This association is difficult to interpret. It is well-known that obese dialysis patients have an improved survival in contrary to what is seen in the general population^27^. This phenomenon is termed reverse epidemiology and has quite a few potential explanations^28^. The most persuasive states that fat mass in dialysis subjects is more a marker of good nutritional status than a risk factor for atherosclerosis and cardiovascular events. Probably, the higher content of n-3 PUFA in obese patients follows the same pattern, being a reflection of better nutrition. However, it might be that the improved survival of obese dialysis subjects is, to some extent, associated with the higher n-3 PUFA content found in these patients.

The major limitation of our study is the modest size of the studied group. However, the differences in the n-3 PUFA levels between dialysis patients and controls were so evident that even with these numbers they turned out to be strongly significant. Furthermore, medications were not considered in the analysis, although the impact of drugs on n-3 PUFA is of limited importance. Finally, food intake was not assessed. Therefore, it is impossible to state whether the observed differences in n-3 PUFA profile between dialysis patients and controls are due to food habits and/or restrictions or to other factors.

In conclusion, the present study confirmed the low n-3 PUFA content in dialysis patients, a finding complemented by evaluation of plant-derived ALA. These low levels further decrease with time on dialysis. Estimating the association between n-3 PUFA composition in dialyzed patients and their prognosis is essential as it can be therapeutically targeted through supplementation. Strong associations between n-3 PUFA and fat mass might contribute to improved prognosis of obese dialysis patients, following the reverse epidemiology phenomenon, a finding that requires confirmation in larger patient cohorts, and verification as to its clinical significance.

## Disclosure statement

The authors report no conflicts of interest. The authors alone are responsible for the content and writing of this article.
